# On the ethics of withholding and withdrawing medical treatment

**DOI:** 10.1186/2049-6958-9-39

**Published:** 2014-07-16

**Authors:** Massimo Reichlin

**Affiliations:** 1 Faculty of Philosophy, University Vita-Salute San Raffaele, Milan, Italy

**Keywords:** Slippery slope, Voluntary active euthanasia, Withdrawing, Withholding

## Abstract

A general rationale is presented for withholding and withdrawing medical treatment in end-of-life situations, and an argument is offered for the moral irrelevance of the distinction, both in the context of pharmaceutical treatments, such as chemotherapy in cancer, and in the context of life-sustaining treatments, such as the artificial ventilator in lateral amyotrophic sclerosis. It is argued that this practice is not equivalent to sanctioning voluntary active euthanasia and that it is not likely to favour it.

## Background

End-of-life situations are among the most prominent areas of controversy in contemporary bioethics. To this day, very few countries have taken the radical approach of accepting the direct termination of life by a medical practitioner. Most countries stick to the traditional interdiction of voluntary active euthanasia. This makes it very much likely that controversy will continue to centre on the practice of withholding and withdrawing medical treatment, with particular reference to life-sustaining treatments [1]. To withhold or withdraw some forms of treatment, in fact, is the simplest way to defend patients from possibly unwanted negative consequences of life-prolonging medical technology, especially when the patient’s quality of life lowers dramatically.

Countries such as the US have an established tradition of legal experience with this sort of medical decision-making: acceptable standards are clearly defined and widely accepted, both for competent and incompetent patients [2]. In Italy, on the contrary, for several cultural reasons, this practice has not taken root yet: it is not that decisions to forego medical treatments are in fact not taken in Italian ICUs, rather that there is no widely agreed and publicly declared policy for making them. Evidence of this lack of agreement is provided by the very large public controversy raised by two cases that have shaken the public’s conscience in recent years, both dealing with matters of withdrawing medical treatment at the end of life. In the case of PiergiorgioWelby, the artificial ventilator was removed from a patient suffering from lateral amyotrophic sclerosis (LAS), while in the case of Eluana Englaro artificial nutrition and hydration (ANH) were withdrawn from a lady who had been lying in a vegetative state for more than 17 years. Both cases elicited widespread public debate and charges of sanctioning medical killing were raised against the tribunals that incriminated neither physician involved.

The public discussion on the Englaro case was dominated by the somewhat bizarre question concerning whether ANH should be considered a form of therapy; in fact, the lower courts that had refused several times to grant the removal of ANH, had declared that only those medical treatments that count as therapies may be subject to a judgment of appropriateness and be legally refused by a competent patient (or by an incompetent one’s legal attorney). And most of the opposition to the Supreme Court’s final ruling accepting the removal of ANH, provided that this was the ascertainable will of the now irreversibly unconscious patient, centered on the somehow confused argument that this was not a case of deciding on the most appropriate use of medical technology, but of simply providing food and water to an handicapped person.

The Welby case was not complicated by these highly questionable assumptions: it clearly concerned life-prolonging medical treatments and the limits of their proper use, along with the role to be acknowledged to the patient’s autonomy in the decision-making process. The patient had been suffering from LAS for almost fifty years and was quadriplegic from twenty-five; he was competent, adequately informed and firmly decided to have his will respected. The physician who accepted to induce ‘terminal’ sedation on him and to switch off the ventilator was found by the court to have acted in agreement with the professional and legal standards; critics, however, have stressed the fact that the patient’s explicit intention to terminate his own life made the case very similar to one of suicide, and the willing cooperation by the physician made it a case of voluntary active euthanasia. The case, therefore, offers the occasion to discuss the meaning and justifiability of the distinction between actively terminating a patient’s life and foregoing medical treatment, as well as of the distinction between withholding and withdrawing treatments. It does so, moreover, in the context of a neurodegenerative disease such as LAS, in which questions concerning the possible limits of the duties to prolong life in the face of a rapidly declining quality of life arise with particular force.

### The moral irrelevance of the distinction between withholding and withdrawing medical treatment

Let’s start with a very general question. What is the ethical rationale for not doing whatever is technically possible to do in patients near the end of life? The general and trivial answer is that not everything that we can do thanks to biomedical resources promotes the best interests of patients, nor is for the patients’ good. This answer, of course, should be made more precise by engaging in a definition of the patient’s good, or of her best interests: how can these notions be spelled out and who is competent to define them? These are notoriously difficult problems, but it is enough to say that, whatever answer one may give to these questions, one thing is clear: that the patient’s good is a complex notion, which cannot be reduced to its mere biomedical dimension [3]. It is one thing to say that whatever promotes the patient’s biomedical interest, as ‘objectively’ defined in handbooks of medical therapy, is a relevant aspect of the patient’s good, and quite another thing to say that, in every situation, the latter notion can be identified with the former. This is clearly not so: and this non-coincidence of the patient’s medical interest with the patient’s overall good is in fact the source of all ethical dilemmas in end-of-life medicine and the basic rationale for accepting the withholding and withdrawing of some forms of medical treatment.

This general approach has long been accepted and practised in the context of oncology. There are several situations, in different forms of cancer, in which the patient, after the failure of first line treatments, faces different prospects: either she accepts to initiate some second- or third-line chemotherapy, with perhaps small chances of real benefit, or she accepts that nothing more can be done by way of causal treatment and switches to some form of palliative care. The ethical rationale of this widely accepted strategy is that no one can be coerced into accepting everything that can possibly confer some medical benefit, however small: it is up to the patient, who will decide with the help of her physicians and relatives, to say whether she wants to be a ‘fighter’ till the end, or prefers to live peacefully at home (or in a hospice) the time that is left. This choice inevitably has to do with several subjective factors: it depends on the values on which the patient’s life was built, and perhaps even more on her character and her general attitude towards life and its difficulties; it also depends on the quality of the care the patient is receiving, on how supportive her environment is, on whether there are familiar or personal reasons for enduring as much as possible. In accepting the patient’s decision in a situation like this, the physician is respecting her capacity to decide for herself what is the best way to conclude her life: the physician, that is, does not impose a predefined view of the ‘good death’, and refrains from excessively medicalising the final events of the patient’s life.

In cases like this, there is no rationale for attributing any moral weight to the distinction between withholding and withdrawing medical treatment. Let us imagine two situations: in the first, the patient simply refuses to undertake one more line of chemotherapy and decides to switch to palliative care. This is clearly acceptable, on the basis of the argument just mentioned against imposing unwanted treatment on competent patients. In the second case, the patient accepts to undergo another line of chemotherapy: she believes herself to have sufficient strength to bear the treatment, and perhaps she is also attracted by the idea of contributing to the experimentation of a new combination of drugs that may prove useful for herself and/or for future patients. However, results leave much to be desired. After some time she feels that her strength is leaving her, that side effects are too burdensome and that her willingness to hold on is vanishing: so she chooses to exit the trial and to switch to palliative care. The same rationale previously accepted for withholding chemotherapy is clearly valid in this situation: the patient has tried, but the treatment did not succeed in bettering her conditions, and perhaps even worsened them. In the light of the experience, she has come to believe that her best interests are now served by abandoning the treatment and accepting a non interventionist approach. This is morally acceptable as well, for the only alternative would be to put patients in the difficult situation of having to choose between not accepting a life-prolonging treatment and accepting it with the implicit commitment not to withdraw it in any situation. But this is not defensible, since it would deprive many patients of the benefits of certain treatments, at least for a certain amount of time.

Clearly, when a patient withdraws a treatment that might prolong her life for some time, she accepts that her life will be shorter than it might have been; but to define the ‘patient’s good’ is in fact to strike a balance between different competing considerations, including those concerning the quantity and quality of the remaining life. It is perfectly acceptable to give up some time of one’s life in order to ‘acquire’ some more ‘quality time’ allowing one to prepare herself to die in a peaceful way. It is also evident that the principle governing this kind of medical decision is utterly different from the one sanctioning voluntary active euthanasia (VAE): the rationale for accepting VAE is a moral principle according to which the patient has a right, based on autonomy, to decide when to terminate her life and the capacity to confer to her physician the right to kill her; the rationale for accepting the withholding and withdrawing of medical treatment, on the other hand, is a moral principle according to which the patient has a right to decide the therapies she is willing to accept and those she does not want.

## Discussion

Is this general rationale for withholding and withdrawing pharmaceutical treatment in the context of oncologic disease also applicable to life-sustaining medical technology, such as the artificial ventilator in situations in which the patient is unable to breathe spontaneously? In particular, does the moral irrelevance of the distinction between withholding and withdrawing hold in these contexts as well? One possible reason to deny that the moral irrelevance thesis holds with reference to such life-prolonging treatments as artificial ventilation is the fact that, whereas withdrawing chemotherapy in the oncology case does not have a direct, immediate effect on the patient’s life, and may even produce better overall consequences, withdrawing ventilatory support from a patient suffering from LAS, or otherwise unable to breathe spontaneously, has the direct effect of bringing about the patient’s death [4]. Thus - it could be argued - while withdrawing chemotherapy is acceptable because it does not imply any direct killing of the patient, withdrawing artificial ventilation is impermissible because it amounts to VAE. According to this view, the distinction between withholding and withdrawing becomes morally relevant in this case, because in the former the physician refrains from intervening and cannot be considered the cause of the patient’s death, whereas in the latter the act of turning off the ventilator is the causally decisive factor for the termination of the patient’s life.

This reasoning, however, is far from being convincing. In fact, it relies very heavily on a literal interpretation of the distinction between acts and omissions, assuming that everything that counts as an omission is, ‘by this very *reason’* , acceptable, and everything that counts as an action is thereby impermissible, so long as its effect is the patient’s death. Contemporary discussion, however, has shown that some cases of letting a patient die are just as morally problematic as cases of directly killing: for example, when cardiac surgery is withheld in a newborn whose Down’s syndrome was not detected by ultrasonic scans, it is clear that the treatment would be appropriate and effective, and the intention, in omitting it, is to terminate the baby’s life. Therefore, it cannot be the mere distinction between acting and not acting that makes a moral difference, rather the conditions in which actions and omissions take place. In the case under consideration, not to initiate mechanical ventilation is causally equivalent to terminating its use, since the patient is unable to breathe spontaneously and the ventilator is the only available means to prolong her life. The physician who, accepting an advance directive (or the present refusal by the patient), refrains from initiating the ventilator treatment is thereby accepting that the patient’s death will follow in a short while. What justifies this decision is not the physician’s doing nothing, but the fact that the patient and the physician are agreed that the benefits to be gained by insisting on the treatment do not justify the burdens that it imposes on the patient, considering her situation and quality of life.

Now, if this is a valid rationale for withholding mechanical ventilation in certain cases, it is valid for withdrawing it as well. In fact, the patient and the physician are agreed that the treatment is now imposing more burdens than benefits to the patient and that, after resisting the disease for so long, it is time to surrender to it, for prolonging life in this situation would not be in the patient’s best interest. This is not equivalent to accepting that the patient has a right to the termination of her life and that the physician is justified in killing her; those who claim that *no* moral difference exists between killing and letting die [5-7] fail to see that the principle governing the action in withdrawing ventilatory support is the respect of the patient’s will concerning medical therapy, not the respect of the patient’s decision to terminate her life [8]. This fine-grained distinction was partly obscured in the case of PiergiorgioWelby by the patient’s explicit request of VAE. But the physician who switched off the respirator declared that he was acting upon the moral principle that obligates physicians not to impose unwanted treatments on their patients and denied having practised euthanasia. Such constraint against imposing medical treatments on unwilling patients is also the Constitutional principle to which the physician’s lawyer in fact appealed in front of the Court.

One possible objection to this reasoning is that, assuming that the legalisation of VAE is not desirable, accepting the withholding and withdrawing of life-prolonging medical treatments can all too easily become the first step on a slippery slope eventually leading to the acceptance of medical killing. Slippery slope arguments are notoriously very difficult to assess, for long term consequences are often highly speculative and the causal link between them and what we decide today is not easy to establish. Nonetheless, I believe that these arguments have some weight and that we should act with the utmost prudence in the context of end-of-life decisions. However, it is vital to take into account *all* the consequences of the different options. So, even if we could not exclude a slippery slope towards euthanasia, we should nonetheless evaluate the consequences of not accepting the interpretation offered thus far. These are that life-prolonging treatments would become mandatory and the power to artificially sustain human life would become a sort of technological cage from which patients could never escape. In other words, the result would be to transform technological opportunities into unconditionally binding moral imperatives – something certainly not desirable. If we wish to avoid the ‘technological cage’ result, we have strong reasons to accept the difference between withdrawing life-prolonging treatment and actively terminating the patient’s life. Of course, someone may add that other conditions, in themselves just as miserable, do not depend on technological support for their prolongation. For example, if a patient is quadriplegic but has no need for artificial ventilation, shouldn’t we accept her request to be released from this situation as well? I am not suggesting that to answer in the affirmative may not be a plausible move. I just want to stress that such an answer is not implied by the acceptance of a general principle against imposing unwanted treatment. To decide whether to impose or not a medical treatment is a medical decision that has to do with the physician-patient relationship: to decide whether to kill a patient whose life needs no medical treatment is an altogether different, perhaps ‘existential’ kind of decision.

Sliding from one kind of decision to the other is not impossible. Nonetheless, there is no direct link between accepting the withholding and withdrawing of life-prolonging treatments and accepting VAE. And insisting that the two practices belong to distinct areas of human action, and may be justified by different moral principles, should be effective in keeping them distinct and avoiding any sliding from one to the other.

## Conclusions

In conclusion, I believe we have quite good reasons for endorsing the interpretation of the moral and legal principles governing the use of life-prolonging means that was sanctioned by both Welby and Englaro cases: to grant patients a consistent opportunity to withhold and withdraw all kinds of medical treatments is in fact to confer them a substantial warrant against the unwanted consequences of medical development and may weaken the drive towards the much more problematic option of changing existing regulations concerning the direct killing of patients.

## Competing interests

The author declares that he has no competing interests.
